# The role of shear dynamics in biofilm formation

**DOI:** 10.1038/s41522-022-00300-4

**Published:** 2022-04-29

**Authors:** Erifyli Tsagkari, Stephanie Connelly, Zhaowei Liu, Andrew McBride, William T. Sloan

**Affiliations:** College of Science and Engineering, School of Engineering, University of Glasgow, Glasgow, G12 8LT United Kingdom

**Keywords:** Microbiome, Biofilms

## Abstract

There is growing evidence that individual bacteria sense and respond to changes in mechanical loading. However, the subtle responses of multispecies biofilms to dynamic fluid shear stress are not well documented because experiments often fail to disentangle any beneficial effects of shear stress from those delivered by convective transport of vital nutrients. We observed the development of biofilms with lognormally distributed microcolony sizes in drinking water on the walls of flow channels underflow regimes of increasing complexity. First, where regular vortices induced oscillating wall shear and simultaneously enhanced mass transport, which produced the thickest most extensive biofilms. Second, where unsteady uniform flow imposed an oscillating wall shear, with no enhanced transport, and where the biomass and coverage were only 20% smaller. Finally, for uniform steady flows with constant wall shear where the extent, thickness, and density of the biofilms were on average 60% smaller. Thus, the dynamics of shear stress played a significant role in promoting biofilm development, over and above its magnitude or mass transfer effects, and therefore, mechanosensing may prevail in complex multispecies biofilms which could open up new ways of controlling biofilm structure.

## Introduction

There are many scenarios where scientists and engineers aim to control the structure and function of biofilms that are fixed to a surface in moving stream of water^1–5^. Thus, for example, enhancing the transport of nutrients to the surface is known to promote the growth of biofilms^6–10^. To counter this and limit the thickness of a biofilm, drag from the flow pressure and shear stress is controlled by varying the fluid velocity to induce the detachment of biomass.

Whilst the sloughing of biofilm in response to hydrodynamic forces is undoubtedly a major factor in controlling biomass there are reasons to believe that the role of shear in biofilm formation may be more complex. For example, in multispecies biofilms the diversity of species is affected by shear stress^8^. Single species biofilms have been observed to alter their metabolism in response to increases in shear stress such that they dissipate more energy^11^ and the binding of adhesive proteins that contribute to cell attachment can be stronger when formed under higher shear conditions^12,13^, which might suggest the ability of bacteria to sense and respond to the stress. This potential for mechanosensing has been reinforced in a number of studies where enhanced concentrations of signalling molecules have been observed in single-species biofilms that are attached to surfaces and subject to shear^14–16^ and a quorum sensing response to these molecules is the expression of genes that play a role in adhesion. In addition to these ecological and biochemical reactions to shear, rheotaxis, where bacteria move in directions that are a function of the magnitude and orientation of shear gradients, is thought to be a purely physical response that occurs in both bulk liquids and in the colonisation of surfaces where the magnitude of the wall shear stress is heterogeneously distributed^17^. Many important environmental flows exhibit turbulence, which is associated with the increased mass transfer but also induces, often large, highly variable shear forces; turbulence has been found to promote the aggregation of cells and enhance biofilm formation^18–20^. So, there is good reason to believe that it is not only the supply of nutrients that affects the colonisation and subsequent growth of cells and the production of extracellular polymers (EPS) but also the nature of the fluid shear experienced at the site where the biofilm forms.

Most experimental designs presuppose that it is the fluid flow’s effects on mass transport that dominate the formation and growth of biofilms. In doing so they neglect the fact that wall shear forces may also have an effect that will vary in time and space, for example, fluctuating in response to any coherent flow structures that are propagated. Thus, it is unclear how much the differences in the wall shear stress dynamics contribute to biofilm structure over-and-above the effects of changes in the mass transport. Teasing these effects apart could influence the way we design infrastructure, whether in biofilm-carrying media for biotechnologies^21^ or in antibiofouling strategies^22^. Here we addressed this by measuring the properties of biofilms that accumulated over four weeks on the walls of flow chambers under seven different laminar flow regimes observed over two sets of experiments, summarised in Table 1.

**Table 1:** The dimensions and characteristics of the flow in each of the flow channels.

	Hydraulic diameter of flow channel (cm)	Obstacle dimensions h x b x l (cm)	Average wall shear stress (mPa)	Average flow rate (cm^3^/s)	Average Velocity (cm/s)	Reynolds Number*
First set of experiments						
Steady Flow (SF)	2.6	n/a	2.4	8.3	1.23	300
Small Vortex Flow (SVF)	2.6	0.4 × 1.2 × 2.6	2.4	8.3	1.23	300
Large Vortex Flow (LVF)	4.5	1.2 × 3.6 × 4.5	2.4	8.3	0.41	175
Second set of experiments						
Large Vortex Flow (LVF)	4.5	1.2 × 3.6 × 4.5	2.4	8.3	0.41	175
High Shear Flow 1 (HSF1)	4.5	n/a	3.5	40	1.98	840
High Shear Flow 2 (HSF2)	2.6	n/a	3.5	8.3	1.23	300
Oscillating Flow (OF)	3.2	n/a	2.4	8.3	0.99	300

*Reynolds number assumes a kinematic viscosity, *ν* = 1.06 mm^2^/s.

Note that three realisations of each flow channel were printed and the experiments were conducted in triplicate.

In the first set of experiments, the aim was to compare the biofilms that formed under two flow regimes where mass transfer to the walls was enhanced by passively induced vortices of different sizes, Large Vortex Flow (LVF) and Small Vortex Flow (SVF), with a third where unform steady flow (SF) regime imposes a wall shear stress equivalent to the time-averaged mean for the vortex flows. Each of the three flow regimes was replicated in triplicate flow channels and the volumetric flow rate was the same in all nine experiments. The experiments were run in parallel and fed with the same tap water to ensure that the channels were exposed to similar drinking water microbiota. A variety of different flow regimes could have been selected to enhance the mass transport to the channel walls. We elected to use a von Karman vortex street shed in the lee of a triangular prismatic obstruction siting in the channel^23,24^ for two reasons. The first, and most pertinent to the aims in this paper, is that the vortices impose fluctuations in shear stress on the channel walls. The second was that transport is known to be passively enhanced by vortices^25^, which requires no mechanical mixing or forcing of the flow. This is incidental to the current aim but is important to a longer-term more general goal of developing low energy biotechnologies^26–28^.

In the second set of experiments, we implemented four different flow regimes in triplicate flow channels. As before, all twelve channels were run in parallel and fed with the same tap water to ensure similar microbiota. We repeated the LVF regime in the channel from experiment set 1. Alongside, we used a pump to induce oscillating flow (OF) in an unobstructed channel, such that the oscillating shear stress on the channel wall approximated that of the LVF but with a uniform regime with parallel streamlines and thus, no enhanced transport. The approximate flow was a sine wave with amplitude and period determined by Fourier analysis of the simulated LVF wall shear. In addition, we operated channels with two further steady flow regimes that imposed a wall shear stress that was equivalent to the maximum experienced in the LVF: one where the channel had the same dimensions as before, High Shear Flow 1 (HSF1), which meant that the average volumetric flow rate was significantly higher and; the other High Shear Flow 2 (HSF2) in a narrower channel which allowed the average volumetric flow rate to be the same as in LVF and so the average flux of bacteria and nutrients was the same. Thus, the second set of experiments aimed to disentangle the relative importance of mass transport, oscillating shear, and shear magnitude on the biofilm characteristics.

**Fig. 1:**
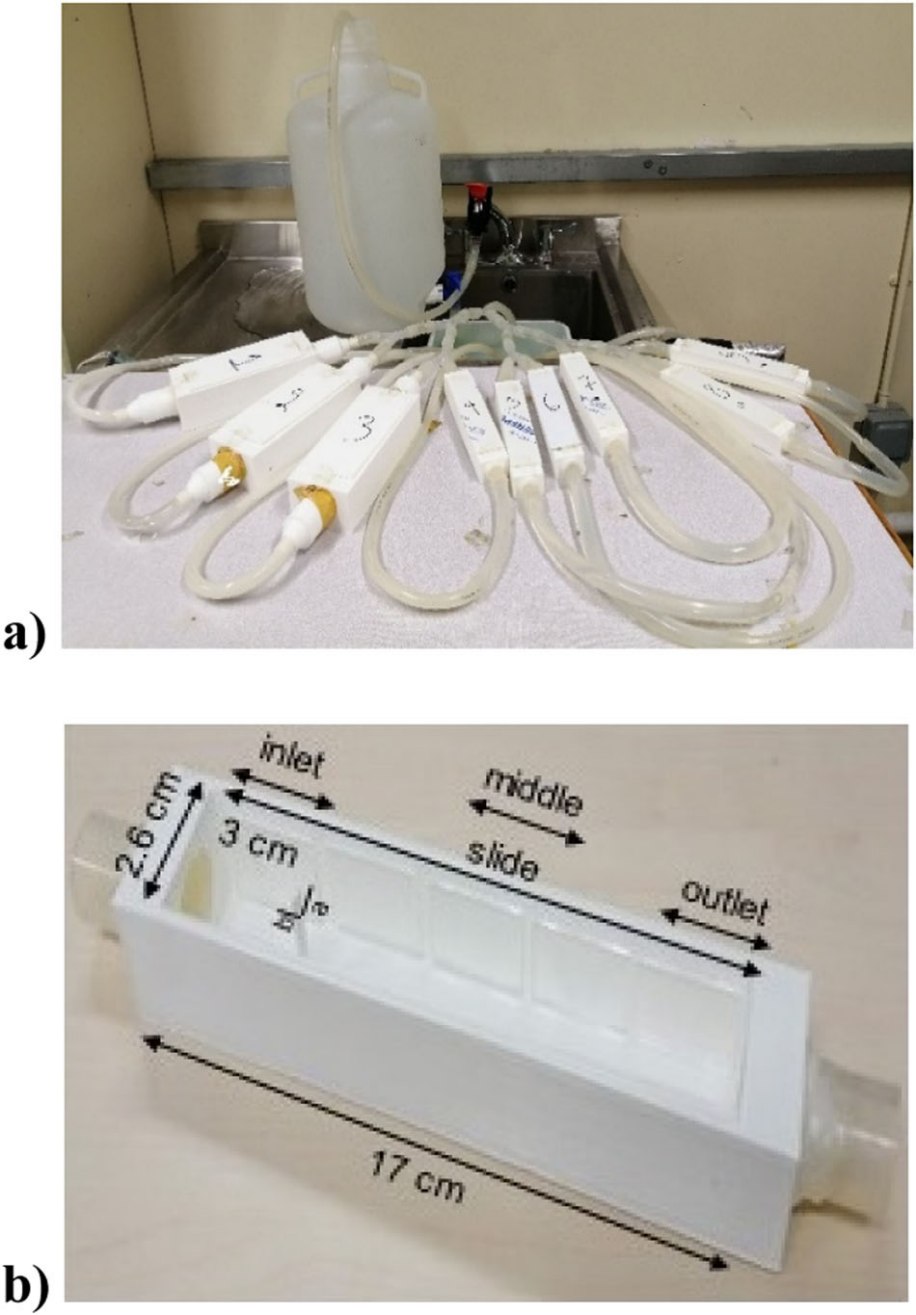
An example of the experimental set-up. **a** The experimental set-up of the first set of experiments comprising of the tap, the water tank, the tubing and valves, the channels (1-3 for LVF, 4-6 for SF and 7-9 for SVF), and the water basin in the sink. **b** The inside of a SVF channel (17 cm length and 2.6 cm width) with the location of the slide sections (inlet, middle and outlet) which are 5 in total of 3 cm length and separate from each other by 0.4 cm, and the position of the triangular object (a = 0.6 cm and b = 0.2 cm) at 3 cm from the entrance.

In our study the design of the channels that produce the desired flow regimes was achieved through a computational fluid dynamics model solving the Navier Stokes equations for a representative horizontal slice through the channel, developed using the deal.II. software library^29^. We then extrapolated the *in-silico* channel geometry from 2-D to 3-D and used a 3-D printer to create the flow channels. An image of flow channels can be seen in Fig. 1.

## Results

**Fig. 2:**
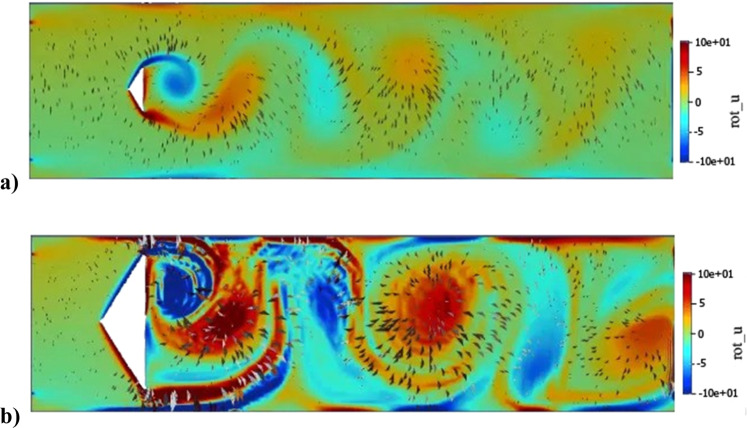
A snap shot of vorticity. **a** SVF experiment. **b** LVF experiment at 41 s. Vorticity (rot_u) is displayed from -10 to 10 s^-1^ based on the bar on the right, in the flow channel calculated by the 2-D computational model. The arrows indicate the direction of flow and the intensity of the vortices.

**Fig. 3:**
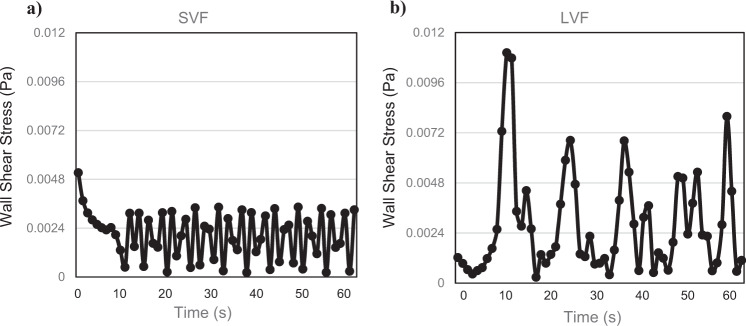
The dynamics of wall shear stress. **a** SVF experiment. **b** LVF experiment. Wall shear stress (Pa) is calculated at a position 6 cm down the channel walls using the 2-D CFD model.

Dimensions, flow regime and environment in the channels. We evolved the geometry of a 2-D channel in the computational model through a process of trial and error until we achieved two distinctly different flow regimes that were amenable to being reproduced experimentally: one with large vortices (LVF) and the other with small ones (SVF). A snapshot of the flow patterns for the two regimes is given in plots of the vorticity in Fig. 2, which show the sequence of counter-rotational vortices associated with the shedding of a von Karman vortex street in the lee of the triangular obstacle. The simulated wall shear stresses underwent an approximately sinusoidal oscillation that varied in amplitude and phase with location and reflected the acceleration and deceleration of water velocity as vortices pass by. Examples of the simulated variation in wall shear stress at a location of 6 cm along both channel walls is given in Fig. 3. The mean wall shear stress for both LVF and SVF was the same, 2.4 mPa. We are simulating a 2-D plane within the 3-D channel and so the predicted amplitude and frequency of oscillations in wall shear stresses are indicative. However, the flow rate and channel dimensions do constrain the time-averaged velocities and so the mean wall shear stress will be accurate. The dimensions for the unobstructed channels SF and OF were selected to give the same mean wall shear, 2.4 mPa and volumetirc flow rate and for HSF1 and HSF2 to have a wall shear equivalent to the maximum of LVF, but with different flow rates (Table 1). The Reynolds numbers, also shown in Table 1, indicate that all flows were laminar. No significant differences (*P* > 0.05) in either the ambient temperature or the temperature of the effluent water was observed over the course of the study, therefore, none of the variance in biofilm characteristics could be explained by temperature (Supplementary Table 1). We verified that the flow patterns in the channels were qualitatively very similar to those predicted the 2-D horizontal plane by releasing thin streams of coloured dye into the flow. Supplementary Fig. 1 gives some sample snapshot images for the LVF where sequences of large vortices that alternate between clockwise and anticlockwise rotations can be observed.

### Biofilm coverage, density and thickness on channel walls

**Fig. 4:**
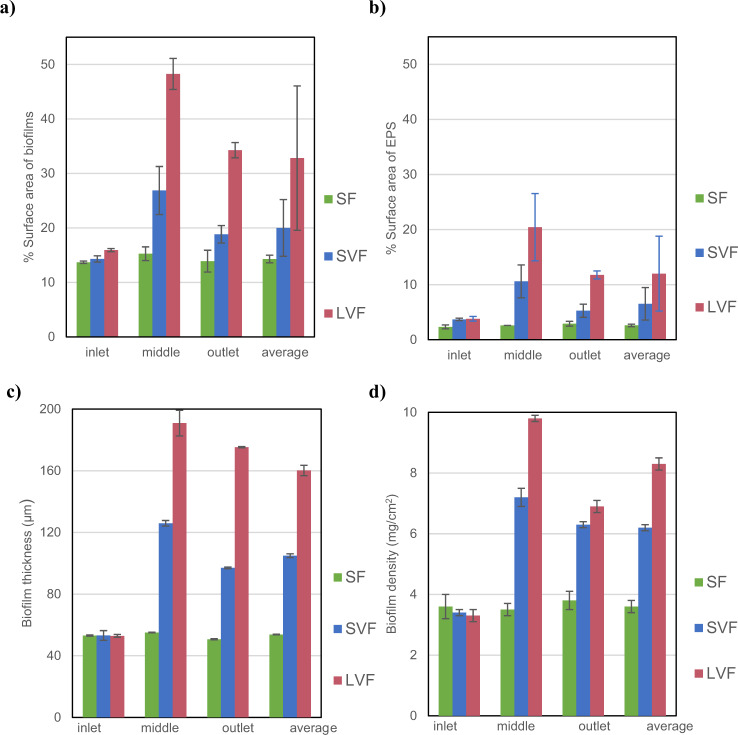
Characteristics of the biofilms that accumulate on the slides on the channel walls for experiment set 1 at three different locations, inlet, middle and outlet, and the average across the whole wall. **a** The percentage of the slide covered with biofilm. **b** The percentage covered by EPS alone. **c** The biofilm thickness. **d** The biofilm density. Note the spatially average density and thickness weighted to the values in areas where the coverage is highest. The error bars in the histrograms represent + /- one standard deviation.

The percentage of surface area covered by total biofilm (cells plus EPS) and EPS were calculated for slides taken from the inlet, middle and outlet of each channel each week during the 4-week experiments and an average across all the slides was also evaluated. In addition, using gravimetry, the spatially average density and thickness of biomass at these locations was determined. The values reported are the averages and standard deviations from three repeats of each flow regime and triplicates of each measurement. The build-up of coverage as the biofilms develop in both experiments is given in Supplementary Fig. 2. The values of the biofilm characteristics at the end of the four-week period for experiment set 1 are displayed in Fig. 4. The vortices only appear in the lee of the obstacles for SVF and LVF and thus the measure of biofilm characteristics on the slides at the inlet, before the obstacles, are the same. The shear stresses will vary even over the surface area of each of the slides, but this was not manifest as a significant difference in coverage in the randomly sampled images from each slide (*p* < 0.05), or indeed from the same slide locations in the triplicate flow channels. In the steady flow (SF), with no obstacle, there is no significant differences (*p* < 0.05) in each of the statistics used to characterise the biofilm between all the slides. for LVF and SVF there is a significant increase that correlates with the onset of the vortices. For all channels, total biofilm and EPS coverage (Fig. 4a, b), biomass thickness (Fig. 4c) and density (Fig. 4d) were consistently ranked highest in the LVF, followed by SVF and then SF. Thus, the degree of mixing induced by coherent flow structures seems to correlate with thicker, denser, and more extensive biofilms, which agrees with the received wisdom that enhanced transport promotes biofilm growth. However, in addition to enhancing the transport of nutrients and bacteria the vortices also impose a variable shear which may also influence the biofilm coverage. Indeed, for both LVF and SVF the middle section of the channel wall, which experiences the greatest variance in shear, had the highest values for all of these biofilm characteristics.

**Fig. 5:**
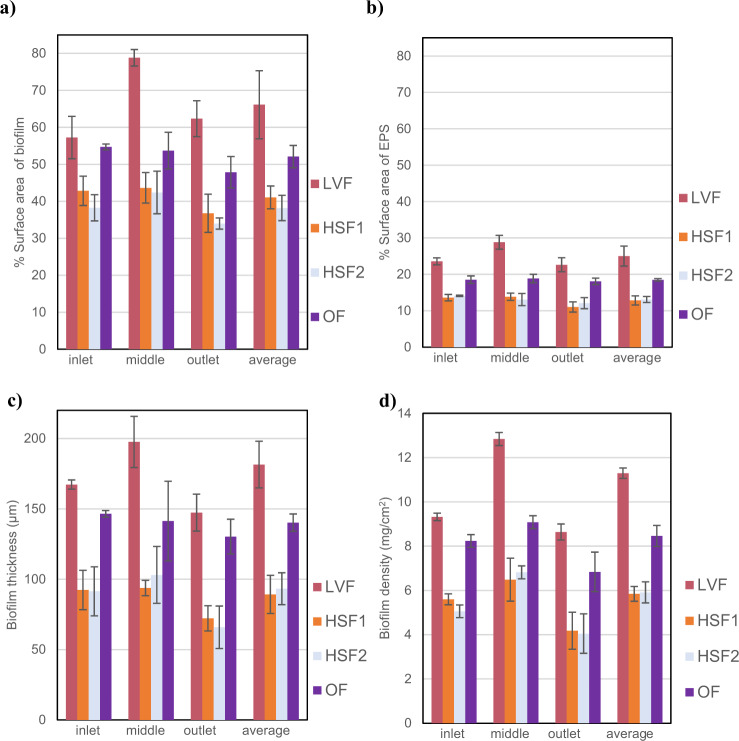
Characteristics of the biofilms that accumulate on the slides on the channel walls for experiment set 2 at three different locations, inlet, middle and outlet, and the average across the whole wall. **a** The percentage of the slide covered with biofilm. **b** The percentage covered by EPS alone. **c** The biofilm thickness. **d** The biofilm density. The error bars in the histrograms represent + /- one standard deviation.

In the second set of experiments, all of the measures of biofilm characteristics were, again, found to be highest for the LVF. The coverage of biofilm (Fig. 5a) and the EPS in isolation (Fig. 5b) measured for the LVF was significantly higher in the second set experiments than in the first (Fig. 4), even though the experimental setup was identical, however, the thickness (Fig. 5c) and density (Fig. 5d) were similar between the two experiment sets. This merely reflects the fact that the density of bacteria in the tap water that fed the two experimental sets was significantly different: means 5.2 × 10^5^ and 8.1 × 10^5^ cells/ml and standard deviations 0.7 × 10^5^ and 1.1 × 10^5^ cells/ml for experiment sets 1 and 2 respectively. The higher density of bacteria in the second experimental set may reflect the fact that the first and second sets of experiments were conducted at different times. The temperature (Supplementary Table 1) of the water did not change significantly and we can only speculate that the community composition, or subtle changes in the water chemistry or, perhaps, the fact that university campus had been largely abandoned between experiments due to Covid 19 restrictions and thus the water in the supply system would have been more stagnant than normal could explain the differences. However, conditions for all experiments in set 2 are identical and so the influx of biomass and substrates was the same, which means comparisons between flow regimes in the set can be drawn. From Fig. 5 it is clear that the LVF, where we have both enhanced transport and oscillating shear, produces the biofilm with the greatest coverage, thickness and density and there is a step up in these measures on the slides downstream of the obstacle where the vortices persist. With the uniform flows, OF, HSF1 and HSF2, there is no change in the measures with location on the channel wall. The unsteady, oscillating flow, OF, had the second-highest measures of all biofilm characteristics and the pair of higher uniform steady flow experiments, HSF1 and HSF2, have the lowest measures. The small differences between the two uniform steady high shear channels (HSF1 and HSF2) were not significant (*P* > 0.05). Thus, the higher mass flux of bacteria and nutrients in the HSF1 did not significantly increase the accumulation or growth of cells over that of HSF2. In addition, it was only the LVF experiment that, like experiment set 1, showed variation in coverage and biomass with distance along the channel. As a crude summary of the relative importance of the various flow regimes across all biofilm characteristics (thickness, coverage and density) we calculated their values as a percentage of the LVF values and averaged these for the slides in the middle and at the outlet of the channels (Table 2). For the biofilms that develops under oscillating flow (OF), where there are no coherent flow structures, the value of this average of coverage, thickness and density is 80% that of LVF. This statistic has a value of 100 for LVF, where the richest biofilms accumulate and 33 where for SF where the steady flow delivers the poorest biofilms. OF, with a value of 80, accounts for $\frac{{100\left( {80 - 33} \right)}}{{100 - 33}} = 70{{{\mathrm{\% }}}}$ of the difference between these extremes, which suggests that the oscillating shear in isolation plays a large part in enhancing biofilm formation.

**Table 2:** Comparison between biofilm characteristics.

	LVF	OF	SVF	HSF1	HSF2	SF
Average over of all biofilm measures as a percentage of LVF (%)	100	80	58	52	51	33

Here we use a single statistic to compare the biofilm characteristics that are produced under the various flow regimes to the biofilm characteristics for the large vortex flows (LVF). The statistic is derived by calculating the thickness, density and coverage for a given flow regime as a percentage of those values measure for LVF in the same experiment set. These percentages are then averaged to give a single value for each flow regime that encapsulates the extensive biofilm properties, and which can be used in the broad comparison between flow regimes presented in the table.

### Attached and effluent biofilm clump size and frequency

**Fig. 6:**
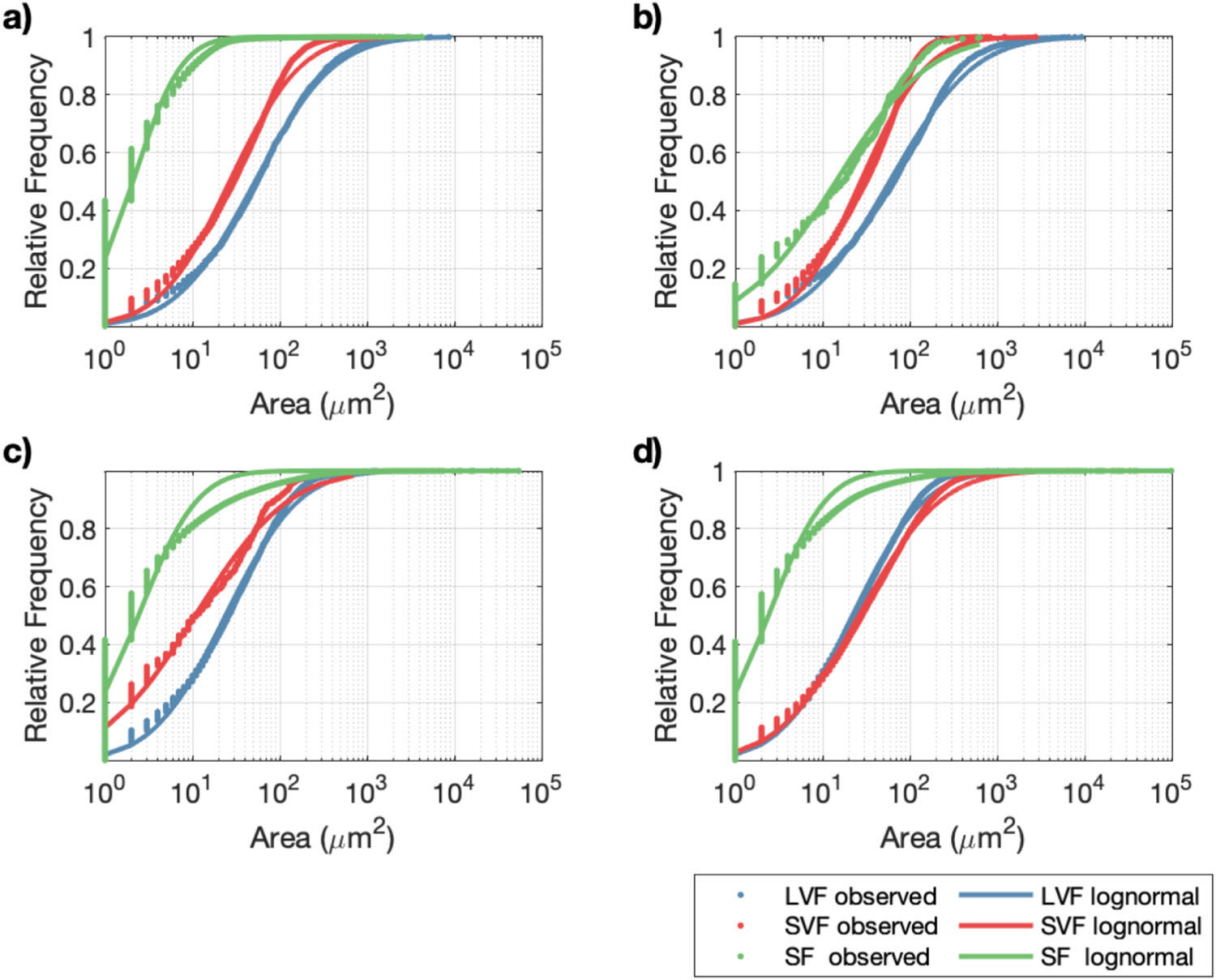
Cumulative frequency distributions of the area covered by individual clumps when imaged by a microscope in experiment set 1 at the end of 4 weeks. **a** biofilm (cells) on slides in the middle section of the channel. **b** EPS only on slides in the middle section of the channel. **c** biofilm on paper through which samples of the channel effluent was filtered. **d** EPS only on paper through which samples of the channel effluent was filtered.

In characterising the biofilms, bacterial cells and EPS that adhered to the surface of the slides on the channel walls were stained separately with two different dyes. Microscope images revealed that both the EPS and the biofilm formed distinct clumps that usually, but not always, coincide; often smaller groups of cells were imaged with no associated EPS. We collated the size of cell-clumps and of EPS clumps from 35 randomly located microscope images on the slide of each channel and have presented them as a cumulative frequency distribution for experiment set 1 (Fig. 6). We have successfully fit lognormal distributions to the data for each experiment, which suggests some common underlying mechanisms are at play, albeit with differences between experiments. Figure 6a shows that the of clumps of biofilm cells for the SF conditions are not only on-average smaller, but their distribution is far more skewed towards small values than for LVF and SVF. There is little apparent difference in the distributions between LVF and SVF, however, the log-scale masks the fact that the mean clump size is smaller for SVF (Table 3). For the EPS component of the biofilm (Fig. 6b) there is less skew towards very small clumps in SF, which would indicate that the small clumps of biofilm that are by far the most abundant on the channel walls under steady flow (Fig. 6a) are dominated by small groups of cells with little associated EPS. There are significant differences (*P* < 0.05) in mean clump sizes of EPS between all experiments: with LVF greatest, followed by SVF and then SF (Table 3).

**Table 3:** The mean and standard deviation of the distribution clumps sizes of stained Biofilm Cells and EPS attached to the channel wall and free-floating in the effluent.

a)								
Clumps Imaged	SF	SF	SVF	SVF	LVF	LVF		
Statistic	Mean	St. Dev.	Mean	St. Dev.	Mean	St. Dev.		
Attached Biofilm Cells (μm^2^)	5.1	29	54	80	164	82		
Attached EPS (μm^2^)	23	28	34	58	55	92		
Free-floating Biofilm Cells (μm^2^)	41	65	48	66	186	68		
Free-floating EPS (μm^2^)	22	54	42	93	80	99		

b)								
Clumps Imaged	HSF1	HSF1	HSF2	HSF2	OF	OF	LVF	LVF
Statistic	Mean	St. Dev.	Mean	St. Dev.	Mean	St. Dev.	Mean	St. Dev.
Attached Biofilm Cells (μm^2^)	64	200	72	206	110	361	273	1600
Attached EPS (μm^2^)	147	863	138	576	158	950	168	1700
Free-floating Biofilm Cells (μm^2^)	72	441	66	407	120	550	186	1100
Free-floating EPS (μm^2^)	168	1400	152	1600	410	1700	458	1900

**a)** Experiment set 1. **b)** Experiment set 2.

Samples of free-floating biofilm that had either sloughed off the channel walls or passed directly through the channel (Fig. 6c) were collected by filtering effluent samples and imaging biofilm cells and EPS on the filter paper in the same way as for the channel wall slides. Figure 6c, d shows the distribution of clump sizes of biofilm cells and EPS alone, respectively. For the distributions of clumps of biofilm cells the skew towards small clumps is most pronounce for SF, followed by SVF and LVF. The mean and variance followed a similar ranking (Table 3a). For the EPS only we see a similar distribution as the biofilm for SF and this suggests that small clumps of biofilm and EPS dominate the effluent. From Fig. 6d there appears to be little difference in the distribution of EPS between LVF and SVF, which is borne out by the statistics in Table 3. The skew towards small clumps in LVF and SVF is less for EPS than the biofilm cells, which suggests that any EPS that sloughs off the walls and into the effluent is in larger clumps than cells.

In the second set of experiments we again saw that the distribution of clump sizes could be modelled by a lognormal distribution. However, the skew towards very small clumps was not as apparent and the plotted cumulative distributions (Supplementary Fig. 3) appeared to be very similar. However, the plots belie significant difference in the statistics of clump sizes (Table 3b). They show that a hierarchy of clump sizes exists, which holds for both biofilm cells and EPS and on the channel walls and effluent: largest clumps in LVF, followed by OF and then the two high shear flows (HSF1 and HSF2). The clump sizes for LVF were significantly higher than in experiment 1, which merely reflects the fact that there is a higher density of cells in the tap water. Indeed, the free-floating EPS has the largest mean clump sizes and highest standard deviation, which suggests the large pieces of EPS are either entering the channel in the tap water or are being sloughed off the channel walls.

## Discussion

Over many years the body of knowledge on how flow conditions shape biofilms^7,30–32^ and how that morphology of biofilms feedback to influence flow regimes has steadily grown. Despite the complexities, a consensus has emerged on some of the important factors. There is now no-doubt that the transport of nutrients, such as carbon and oxygen, to the biofilm surface and their subsequent diffusion in the biofilm has a profound effect, not only on the biomass, but also on the physical structure of biofilms^33^. Our results unsurprisingly reiterate these findings. By passively imposing mixing by inducing vortices in the lee of an obstacle we see that, for the same mean flow rate, the magnitude of the vortices is correlated with the thickness, density and coverage of the biofilm attached to slides on the flow chamber walls. The decision to passively induce flow structures to enhance transport rather than by mechanical controlling the flow was motivated by the goal of low-energy control of biofilm formation in engineering settings, such as biofiltration technologies.

There is a growing recognition that the influence of the forces that hydrodynamics impose on a biofilm on its structure and growth might be more complex than first thought. It has long been established that as fluid velocities increase so does the probability that pieces of biofilm are eroded and entrained into the fluid flow. There is a tacit distinction made between erosion, which refers to small particles, and sloughing, which refers to larger particles. The wall shear stress at the boundary between fluid and the biofilm is often used as an explanatory variable in empirical relationships and in physically-based mathematical models for the onset of erosion events. Whilst shear stress will have an impact on erosion, its magnitude is typically an order of magnitude lower than the normal drag forces caused by pressure on components of the biofilm that protrude into a flow field^16^, yet they are correlated and so shear stress is essentially used as a surrogate for the combined hydrodynamic forces. However, especially in the case of sloughing larger chunks of biofilm, drag and other more subtle effects like the onset and growth of unstable perturbations^34^ may play a more important role than the absolute magnitude of shear stress.

Notwithstanding the subtleties of using shear stress as an index to the mechanical forces on a biofilm, there is growing understanding of potential role of mechanosensing on biofilms. This phenomenon, where a cell’s biochemistry responds to mechanical forces, is vital to the functioning of eukaryote cells in multicellular organisms and has recently been shown to prevail in prokaryotes. Both drag and shear-gradients in fluids have been implicated in this^16^. Thus, the production of signalling molecules such as cyclic-di-GMP^14^ in response to mechanical forces as *Pseudomonas aeruginosa* adhere to a surface has been observed, which goes on to induce the expression of genes that are ultimately associated with biofilm formation, such as the enhanced production of EPS. The expression of these signalling molecules has been observed at stresses significantly below 0.002 Pa^14^, which is the average shear stress in calculated for our experiments.

These biochemical responses to shear have been observed primarily in single species, *Pseudomonas aeruginosa*, biofilms. If they are more generic and are prevalent in environmental bacteria then the role of fluid shear gradients normal to the surface may well be two-fold: creating shear and drag forces that erode the biofilm surface especially where the surface topology is complex; whist at the same time imposing average small shear stresses across biofilm surface that are sufficient to induce signalling responses that lead to the enhancement of factors to strengthen the biofilm, increase its adhesion to the surface and retain and grow biomass. It is only the former that routinely appears in conceptual and mathematical models of biofilms. The relative importance of the latter in dictating the ultimate biomass and structure of multispecies biofilms has not previously been assessed.

Our experiments have gone some way to resolve this. In the experiments where vortices are induced there is the opportunity for three processes to shape the biofilm: the enhanced transport of nutrients and planktonic bacteria to the channel walls caused by the vortices mixing the flow; biofilm production and strengthening in response to mechanosensing; and the erosion of biofilm by shear stress and drag forces. In the remaining experiments, the enhanced transport through mixing is removed but various aspects of the shear regime are retained. Thus, only mechanosensing, laminar boundary layer effects and erosion are at play. In all but one of these, the mean flux is the same and so the average mass transport of bacteria and nutrients into the channel is the same. Where the flow is steady (SF) with a constant shear stress that is the same as the mean shear in the large vortex flow (LVF) the biofilm is at its least dense, covers the least area and is thinnest; on average all these measures are approximately 33% of those for LVF. With steady flows (HSF1 and HSF2) where the shear is increased to approximately the maximum experienced under the large vortex flows the measures were approximately 55%.

In the conventional conceptual model of biofilm formation one might have expected the steady flow regimes with higher wall shear stresses to have less biomass because erosion and sloughing would be higher. The fact that the biomass is greater under higher shear implies that biomass growth or accumulation is sufficiently enhanced to overcome the erosion. This could be the result of a physiological response in certain species or by mechanically induced selection of fast-growing adhesive species^33^ or any increase in the local boundary diffusive mass transfer for nutrients in response to the increased shear gradient or a combinations of these. For the uniform unsteady flow regime where the water velocity is controlled to deliver the same shear at the channel walls as the large vortex flow but with parallel streamlines (OF) then the biofilm measures are, on average, approximately 80% of those for LVF. So only 20% of the values for biofilm thickness, density and cover can be attributed to the mechanical mixing cause by the large coherent flow structures. Furthermore, since the mean shear stress is equivalent to that for steady flow regime (SF) and significantly less than for HSF1 and HSF2 it suggests that the dynamics in the shear are more important in promoting biomass than the absolute magnitude. For eukaryote cells there is both experimental and theoretical evidence to suggest that the dynamics of loading applied to cells affects the material properties of their extracellular matrices^35^ and there are suggestions that similar processes occur in bacteria^16^. Our results are for the whole biofilm rather than individual cells, nonetheless they do seem to support these ideas on fluctuating shear being an important factor in producing robust biofilms.

The biofilms attached to the chamber walls and those collected in the effluent appear as spatially distinct clumps of cells and EPS that usually coincide. Whilst these clumps assumed a wide variety of shapes, they could be crudely characterised by their area. For all experiments the distributions of clump areas, particularly on the channel walls, could be modelled using a lognormal distribution. The lognormal distribution can often be used to describe the distribution of biological entities, especially where they are subject to similar but independent growth^36^. Despite its long-standing prevalence, convincing mechanistic derivations of the lognormal distribution are lacking for most biological applications and, to-date, it has not been used to describe the distribution of clumps or microcolonies in a biofilm. It is, however, deployed in chemistry to describe the distribution of grain sizes in crystallisation processes and a self-consistent mechanistic model for a lognormal grain-size distribution of Si films has been derived based on random nucleation and subsequent growth^37^. The maturity of the film is reflected in the skew of the lognormal distribution towards smaller crystals and is controlled by the relative rates of nucleation to growth.

Biofilms form through the nucleation (or deposition) and subsequent growth of microcolonies and thus there is a direct analogy with the crystal growth model. Therefore, the fact that the skew towards small clumps is greatest for the SF case and decreases through SVF and LVF in experiment set 1 and moves, more subtly, from HSF1 and HSF2 through OF to LVF in experiment 2 potentially reflects the acceleration in the growth of biofilm colonies in comparison to their deposition rate. Perceiving a biofilm as a collection of microcolonies, that eventually merge, may offer a way of modelling biofilms by explicitly simulating the process in a representative sample of different sized microcolonies.

More generally, our results suggest that the dynamics of shear stress affects the structure of biofilms in ways that have been hitherto neglected. Controlling shear dynamics, by inducing and controlling non-steady flows may offer an additional means to regulate biofilms in a wide range of industrial and medical settings.

## Methods

### Computational model

A 2-D model was developed in C + + using the open-source finite element method library deal.II.^29,38^. The model solves the incompressible time-dependent Navier-Stokes equations^39^ to describe the flow around an obstacle for a viscous fluid. The model simulated the flow in a section of a 17 cm long section of channel. No-slip boundary conditions were used for the channel and obstacle walls. We make the reasonable assumption that under laminar flow conditions the biofilm is too thin to have any perceptible effect on flow patterns. A parabolic velocity profile associated with uniform flow with a predetermined mean velocity (i.e. Poiseuille flow) was assumed for the horizontal component of the velocity at the upstream boundary. The vertical component of the velocity was assumed to be zero at both the upstream and downstream boundaries. In addition, the pressure is imposed to be zero at the downstream boundary. The inlet velocity and the dimensions of the triangular obstacle were adjusted until the desired vortex street was obtained. ParaView 5.6.0-RC2, an open-source software package, was used to visualise the vorticity profile within the channel. Microsoft Excel software was used to plot the numeric output of the model to show the distribution of wall shear stresses with time at fixed positions within the channel.

### From model to 3-D printed flow channel

To extrapolate the model to a 3-D print we ‘extruded’ the design in z-direction to an effective channel depth equal to the y-axis dimension for each experiment using Blender software to generate the 3-D design for prototyping. The 3-D model comprised conical inlet and outlet ports on the Z-X face of the channels. The internal diameter of the inlet and outlet port at the entry / exit from the channel was designed as a minimum of 75% surface area of that plane to limit the potential for entry / exit effects on the flow in the channel. The prototype flow channels were printed using the Ultimaker 3 benchtop printer (3-DGBIRE, unit 5 East Reading Retail Centre, Reading, UK) using polylactic acid, a non-toxic print material.

### Model confirmation by flow visualization

To determine that the flow predicted in silico was established in vivo in the printed flow channels dilute black and purple food colouring was introduced to the flow via two hypodermic needles just upstream of the obstacle. The dye was driven through the needles from external chambers under the minimal head gradient possible. Supplementary Fig. 1.

The motion of the dye was recorded using a copy stand (QH-L082 copy light stand, Qihe, Zhejiang, China) and a camera (FinePix Fujifilm HS50EXR, FUJIFILM UK Limited, Bedford, UK).

### Fourier analysis and uniform flow conditions

A Fourier analysis of the simulated shear stresses from the CFD model of LVF flow revealed that 90% of the variance could be captured by a sine wave with an average of 2mPa and amplitude of 1.5 mPa and a period of 2.5 s and thus minimum and maximum wall shear stresses of 0.5 and 3.5 mPa respectively. To achieve this sine wave of shear in the OF experiment required us to deliver flow with an average volumetric flow rate of 8.3 cm^3^/sec, and amplitude of 6.1 cm^3^/sec and period 2.6 s and cross-sectional dimensions in Table 1. A 730 DuN/RE Watson Marlow peristaltic pump was used for the OF experiment. The HSF1 and HSF2 experiments reproduced steady shear equivalent to the maximum and mean of the raw simulated LVF shear time series using volumetric flow rates 40 cm^3^/sec and 8.3 cm^3^/sec respectively; again channel dimensions are in Table 1.

To enable observation of the biofilm formed at the wall of the channel at the end of the experiment, clear cast acrylic slides (Hobarts: Design & Technology Supplies, West Malling, UK) were inserted in each channel along both walls on the Z-Y plane. Each slide was laser cut (Full Spectrum Laser LLC, Las Vegas, US) into 5 sections of 3 × 2 cm (length × height) for all experiments. The sections were detached from the main body of the slide after the 4-week duration of the experiments for biofilm analysis. Henceforth, the sections are reported as: “inlet” which sat before the location of the object at 3 cm in the channel, “middle” is the one after the location of the object in the middle of the channel from 6 to 9 cm, and “outlet” is the last section close to the outlet of the channel.

Experimental set-up. The flow channels were operated under continuous flow of tap water for a 4-week period with the tap water acting as both microbial seed and nutrient supply for biofilm formation. Water was continuously delivered to each flow channel via tubing by maintaining a constant head of 50 cm in a 22 L communal overflow reservoir. Constant head in the overflow reservoir was maintained by continuous inflow of tap water at a feed rate of 100 cm^3^/sec whilst the cumulative outflow to the 9 flow channels was 75 cm^3^/sec for the first set of experiments, and at a feed rate of 200 cm^3^/sec whilst the cumulative outflow to the 12 flow channels was 195 cm^3^/sec for the second set of experiments. For the OF experiment we used a peristaltic pump so that the oscillating shear stress on the channel wall approximated that of the LVF experiment but with a uniform flow regime with parallel flow streamlines.

The water supply to each flow cell was delivered from the feed reservoir using tubing (inner diameter of 0.8 cm Fisherbrand™, Thermo Fisher Scientific, Inchinnan, UK) with Y-shape connectors used to split the flow as required (Supplementary Fig. 1d). Each flow channel had an independent effluent line comprising the same type of tubing with flow control valves at the end (Teflon straight-through valve with screw lock, Fisherbrand™, Thermo Fisher Scientific, Inchinnan, UK) to accurately control the flow rate. The system was sterilized prior to use using 0.5% sodium hypochlorite and 70% ethanol before the onset of experiments. Again, the analytical description of all cases for both sets of experiments can be found in Table 1.

Physical and chemical monitoring. The experiments operated at ambient temperature. The temperature of the water leaving the flow channels and ambient room temperature was measured at hourly intervals throughout using data loggers (HOBO data logger Pendant Temp/light 64 K, Bourne, US) for the total duration of the experiments. Water temperature was recorded in the communal effluent collection tank in order to test the temperature of water without disturbing the flow in the channels. Air temperature was recorded adjacent to the experimental set up. (Supplementary Table 1) Lastly, the concentration of total chlorine in the tap water was measured at each of the outlets of experiments at the end of every week^19^. The concentration of total chlorine of the tap water was measured immediately after its sampling using the USEPA DPD Method 8167 and a colorimeter (DR 900 Hach, Loveland, CO, USA).

### Biofilms on channel walls

One set of slides in each channel was removed and used for gravimetric measurements to characterize the thickness and density of biofilms^18^ at the inlet, middle and outlet of each channel. The other set of slides was removed to be used for visual analysis of the biofilm by differential fluorescent staining of cells and EPS at the inlet, middle and outlet of the channel.

For the gravimetric characterisation of the biofilm we used the following method^40^. First, we removed the slide, allowed it to drain for 5 min then weighed it. The slide was dried for 24 h at 65 °C in an oven and weighed again. The dried biofilm was then washed off and the weight of the clean slide was measured. Thus, the wet and dry weight of the biofilm, *m*_*w*_ and *m*_*d*_ respectively, were determined. The average values for the wet and dry mass of biofilm that were used for the calculation of the biofilm thickness and density can be found in Supplementary Table 2. In all cases the dry mass makes a significant contribution to the total wet mass. For almost all biofilms in water the combined mass of water and biomass per unit volume is very close to the density of water. Thus, a first-order approximation to the thickness of biofilm, *L*, averaged over the whole surface of the slide is,

$$L = m_w/A\rho ,$$

where A is the area of the slide and *ρ* is the density of water. Stuadt et al. (2204) validated the estimates made in this way using 3D CLMS images. Of course, the biofilm density, *ρ*_*b*_, volume of biomass per unit volume occupied by the biofilm is likely to vary significantly for biofilms that are structured in different ways, and this is routinely approximated^40^,

$$\rho _b = \left( {\frac{{m_d}}{{m_w}}} \right)\rho$$

For the differential staining, the biofilm on the slide sections was fixed with 4% paraformaldehyde^41^ and stained using 10 μg/mL Fluorescein Aleuria aurantia lectin (Vector laboratories, Peterborough, UK) for the EPS^5,42^ and then using 10 μg/mL of 4′, 6-diamidino-2-phenylindole (DAPI) for the cells^18^. The samples were firstly covered with 1 mL of 10 µg/mL Fluorescein Aleuria aurantia lectin (Vector laboratories, England, UK) for 10 min in the dark to stain the lectin-specific EPS glycoconjugates. Then, the samples were covered with 1 mL of 10 µg/ mL DAPI for 20 min in the dark to stain the cells. Fluorescence microscopy was performed using the Invitrogen EVOS FL Auto 2 cell imaging system (Thermofisher Scientific, Renfrew, UK), the AMEPVH001 7.66 × 2.62 cm vessel holders and the UPlanFLN 100X oil immersion objective lens were used for visualisation. The differential staining of cells and EPS allows them to be distinguished in a single image from each field of view. Examples of the raw images are given for each flow regime in Supplementary Fig. 4. The surface area of both the total biofilm and the EPS of the biofilm was then calculated in Matlab^18^. Briefly, at randomly selected points on each slide an image of the cells of biofilm was acquired. Then, at the same location, an image of the EPS of biofilm was acquired. The two images were then combined, and a composite image was created in Matlab. The surface area of biofilm from the composite image, and of the EPS of the biofilm from the EPS image only, was then calculated in Matlab^18^. Specifically, the original microscopic images were firstly converted to grayscale images and then to binary images to separate the biomaterial (the biofilms in one case and only the EPS in the other case) from the background of the microscopic image. After the surface area for all the microscopic images obtained was calculated, it was divided to the total surface area of the microscopic image to finally calculate the percentage of the surface area (%).

Lastly, the frequency and size of clumps of cells and of EPS components from the biofilm was calculated in Matlab. The total number of components in each converted to binary microscopic image was calculated using the bwlabel function. After that, the regionprops function was used to measure the size area of each component in the image. The mean function was then used for the mean area size.

Statistical analysis. All measures were analysed in SPSS Statistics 26 (IBM, USA) using one of the following tests: i. the one-way ANOVA in conjunction with the Tukey’s and Duncan-Waller’s tests, ii. the non-parametric tests of Kruskal-Wallis by ranks test, iii. the Mann–Whitney U test, iv. the Median test in conjunction with the Pearson’s Chi-squared and the Phi and Cramer’s tests, and v. the Kolmogorov–Smirnov test, depending on the fitness of the data to the test assumptions. All calculations were based on the confidence level of 95%, which means that a *P* value lower than 0.05 was considered statistically significant^43,44^.

### Reporting Summary

Further information on research design is available in the Nature Research Reporting Summary linked to this article.

## Supplementary information

Reporting Summary Checklist

Supplemental Material

## Data Availability

All data supporting the findings of this study are available from the corresponding author upon request.

## Code availability

The program is coded based on an open source c++ finite element library and the code is available in a public repository on GitHub: https://github.com/zhaoweiliu/dealii_catmull_clark/blob/master/examples/step-35/biofilm.cc.

## References

[1] Chaudhary DS, Vigneswaran S, Ngo HH, Shim WG, Moon H (2003). Biofilter in water and wastewater treatment. Korean J. Chem. Eng..

[2] Fish, K. E. & Boxall, J. B. Biofilm Microbiome (Re)Growth Dynamics in Drinking Water Distribution Systems Are Impacted by Chlorine Concentration. *Front. Microbiol.* **9**, 2519 (2018). 10.3389/fmicb.2018.02519 PMC6232884 30459730

[3] Suwarno SR (2018). On-line biofilm strength detection in cross-flow membrane filtration systems. Biofouling.

[4] Douterelo I, Sharpe RL, Husband S, Fish KE, Boxall JB (2018). Understanding microbial ecology to improve management of drinking water distribution systems. WIREs Water.

[5] Fish K, Osborn AM, Boxall JB (2017). Biofilm structures (EPS and bacterial communities) in drinking water distribution systems are conditioned by hydraulics and influence discolouration. Sci. Total Environ..

[6] Wasche S, Horn H, Hempel DC (2000). Mass transfer phenomena in biofilm systems. Water Sci. Technol..

[7] Horn H, Reiff H, Morgenroth E (2003). Simulation of growth and detachment in biofilm systems under defined hydrodynamic conditions. Biotechnol. Bioeng..

[8] Battin TJ, Kaplan LA, Newbold JD, Cheng X, Hansen C (2003). Effects of current velocity on the nascent architecture of stream microbial biofilms. Appl Environ. Microbiol.

[9] Battin TJ (2007). Microbial landscapes: new paths to biofilm research. Nat. Rev. Microbiol..

[10] Wasche S, Horn H, Hempela DC (2002). Influence of growth conditions on biofilm development and mass transfer at the bulk/biofilm interface. Water Res..

[11] Zhao M, Cheng L (2014). Two-dimensional numerical study of vortex shedding regimes of oscillatory flow past two circular cylinders in side-by-side and tandem arrangements at low Reynolds numbers. J. Fluid Mech..

[12] Berne C, Ellison CK, Ducret A, Brun YV (2018). Bacterial adhesion at the single-cell level. Nat. Rev. Microbiol..

[13] Thomas W (2008). Catch bonds in adhesion. Annu. Rev. Biomed. Eng..

[14] Rodesney CA (2017). Mechanosensing of shear by Pseudomonas aeruginosa leads to increased levels of the cyclic-di-GMP signal initiating biofilm development. Proc. Natl Acad. Sci..

[15] Sanfilippo JE (2019). Microfluidic-based transcriptomics reveal force-independent bacterial rheosensing. Nat. Microbiol..

[16] Chawla R, Gupta R, Lele TP, Lele PP (2020). A skeptic’s guide to bacterial mechanosensing. J. Mol. Biol..

[17] Secchi E (2020). The effect of flow on swimming bacteria controls the initial colonization of curved surfaces. Nat. Commun..

[18] Tsagkari E, Sloan WT (2018). Turbulence accelerates the growth of drinking water biofilms. Bioprocess Biosyst. Eng..

[19] Tsagkari, E., Keating, C., Couto, J. M. & Sloan, W. T. A Keystone Methylobacterium Strain in Biofilm Formation in Drinking Water. *Water* **9**, 9100778 (2017).

[20] Tsagkari E, Sloan WT (2019). Impact of Methylobacterium in the drinking water microbiome on removal of trihalomethanes. Int. Biodeterior. Biodegrad..

[21] Edel M, Horn H, Gescher J (2019). Biofilm systems as tools in biotechnological production. Appl Microbiol Biotechnol..

[22] Kim EH, Dwidar M, Mitchell RJ, Kwon YN (2013). Assessing the effects of bacterial predation on membrane biofouling. Water Res..

[23] De AK, Dalai A (2007). Numerical study of laminar forced convection fluid flow and heat transfer from a triangular cylinder placed in a channel. J. Heat. Transf.-Trans. Asme.

[24] Chen YJ, Shao CP (2013). Suppression of vortex shedding from a rectangular cylinder at low Reynolds numbers. J. Fluids Struct..

[25] Meis M, Varas F, Velázquez A, Vega J (2010). Heat transfer enhancement in micro-channels caused by vortex promoters. Int. J. Heat. Mass Transf..

[26] Stauch-White K, Srinivasan VN, Camilla Kuo-Dahab W, Park C, Butler CS (2017). The role of inorganic nitrogen in successful formation of granular biofilms for wastewater treatment that support cyanobacteria and bacteria. AMB Express.

[27] Miranda AF (2017). Applications of microalgal biofilms for wastewater treatment and bioenergy production. Biotechnol. biofuels.

[28] Yousra T (2017). Biofilms in bioremediation and wastewater treatment: characterization of bacterial community structure and diversity during seasons in municipal wastewater treatment process. Environ. Sci. Pollut. Res Int.

[29] Arndt D (2019). The deal.II Library, Version 9.1. Numer. Math..

[30] Stoodley P, Cargo R, Rupp CJ, Wilson S, Klapper I (2002). Biofilm material properties as related to shear-induced deformation and detachment phenomena. J. Ind. Microbiol Biotechnol..

[31] Curran SJ, Black RA (2005). Oxygen transport and cell viability in an annular flow bioreactor: comparison of laminar Couette and Taylor-vortex flow regimes. Biotechnol. Bioeng..

[32] Rusconi R, Lecuyer S, Guglielmini L, Stone HA (2010). Laminar flow around corners triggers the formation of biofilm streamers. J. R. Soc. Interface.

[33] Rickard AH, McBain AJ, Stead AT, Gilbert P (2004). Shear rate moderates community diversity in freshwater biofilms. Appl. Environ. Microbiol..

[34] Vignaga E (2013). Erosion of biofilm-bound fluvial sediments. Nat. Geosci..

[35] Elosegui-Artola A, Trepat X, Roca-Cusachs P (2018). Control of mechanotransduction by molecular clutch dynamics. Trends Cell Biol..

[36] Curtis TP, Sloan WT, Scannell JW (2002). Estimating prokaryotic diversity and its limits. Proc. Natl Acad. Sci..

[37] Bergmann RB, Bill A (2008). On the origin of logarithmic-normal distributions: An analytical derivation, and its application to nucleation and growth processes. J. Cryst. Growth.

[38] Bangerth, W., Hartmann, R. & Kanschat, G. Deal.II—A General-Purpose Object-Oriented Finite Element Library. *ACM Trans. Math. Softw*. **33**, 27 (2007).

[39] Guermond JL, Quartapelle L (1998). On the approximation of the unsteady Navier—Stokes equations by finite element projection methods. Numerische mathematik.

[40] Staudt C, Horn H, Hempel D, Neu T (2004). Volumetric measurements of bacterial cells and extracellular polymeric substance glycoconjugates in biofilms. Biotechnol. Bioeng..

[41] Chao YQ, Zhang T (2011). Optimization of fixation methods for observation of bacterial cell morphology and surface ultrastructures by atomic force microscopy. Appl. Microbiol. Biotechnol..

[42] Garny K, Horn H, Neu TR (2008). Interaction between biofilm development, structure and detachment in rotating annular reactors. Bioprocess Biosyst. Eng..

[43] Kim TK (2017). Understanding one-way ANOVA using conceptual figures. Korean J. Anesthesiol..

[44] Brown AM (2005). A new software for carrying out one-way ANOVA post hoc tests. Comput Methods Prog. Biomed..

