# Supplementary material for: The role of shear dynamics in biofilm formation
Source: NPJ Biofilms Microbiomes. 2022 Apr 29;8:33. doi: 10.1038/s41522-022-00300-4 (PMC9055050; doi:10.1038/s41522-022-00300-4)
Supplement: Supplementary file 2 — Supplemental Material [file 41522_2022_300_MOESM2_ESM.pdf]

The role of shear dynamics in biofilm formation.

Erifyli Tsagkari, Stephanie Connelly, Zhaowei Liu, Andrew McBride, and William T. Sloan*

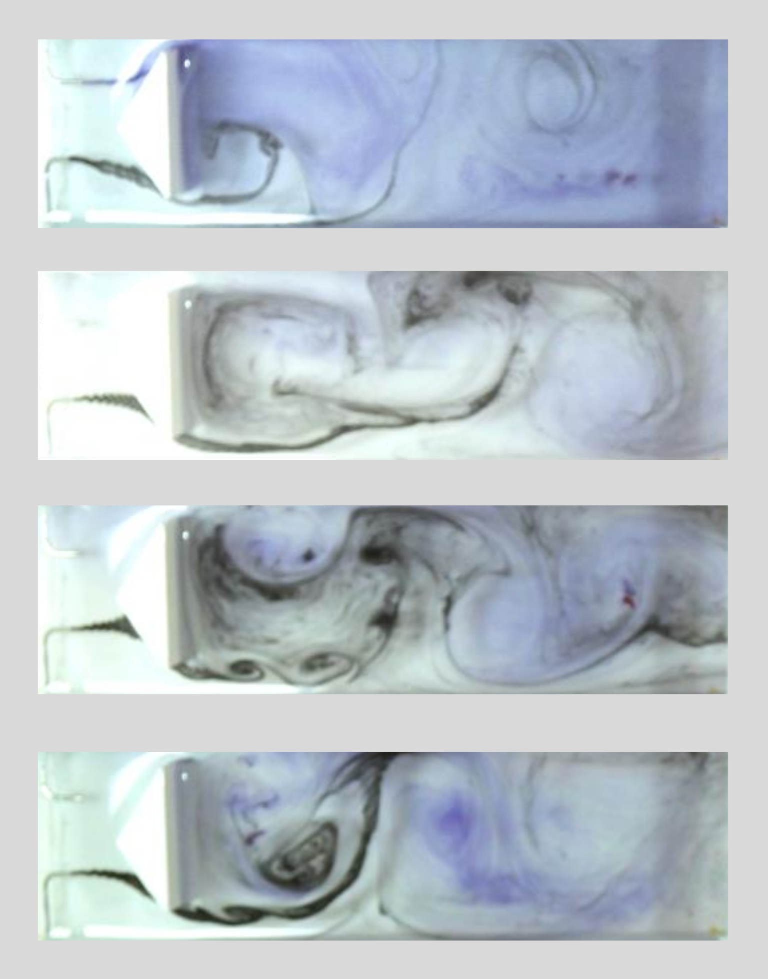

Supplementary Figure 1. Snap shots from the flow visulisation of LVF. They show vortices being shed by the object with alternate clockwise and anticlockwise rotation. The pattern is similar to that simulated by the CFD code.

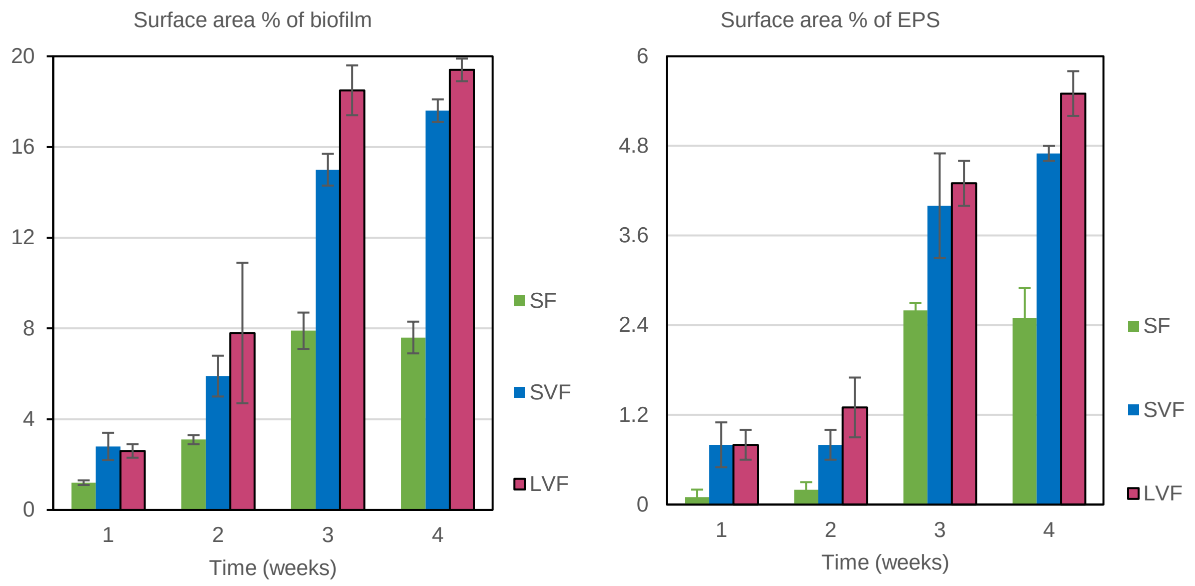

a)

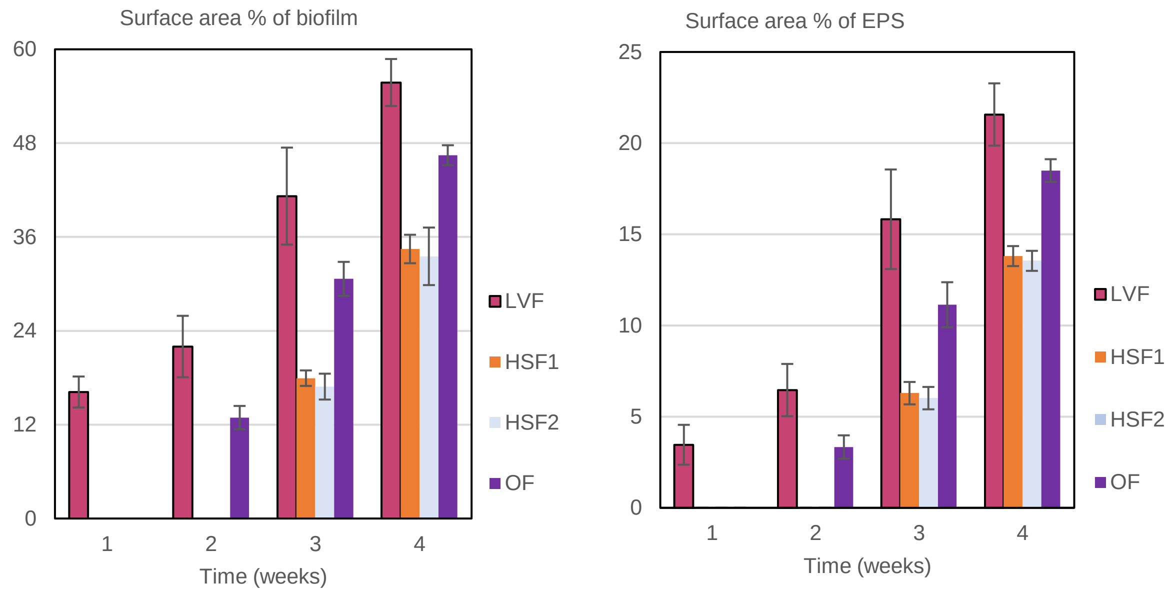

b)

Supplementary Figure 2. Surface area % of the total biofilm (EPS + Cells) and of only the EPS for the material in liquid samples from each of the effluent of **a)** first set of experiments and **b)** second set of experiments. The error bars in the histrogram represent +/- one standard deviation.

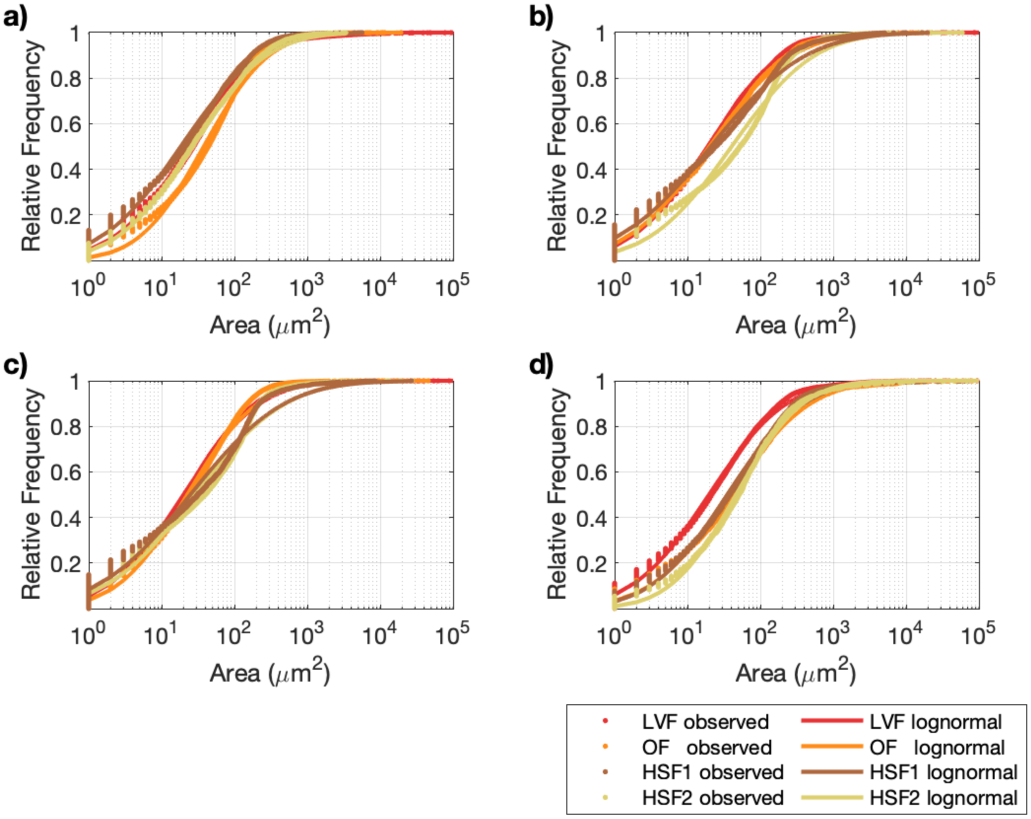

Supplementary Figure 3. Cumulative frequency distributions of the area covered by individual clumps when imaged by a microscope in experiment set 1 at the end of 4 weeks for: **a)** biofilm (cells) on slides in the middle section of the channel; **b)** EPS only on slides in the middle section of the channel; **c)** biofilm on paper through which samples of the channel effluent was filtered; **d)** EPS only on paper through which samples of the channel effluent was filtered.

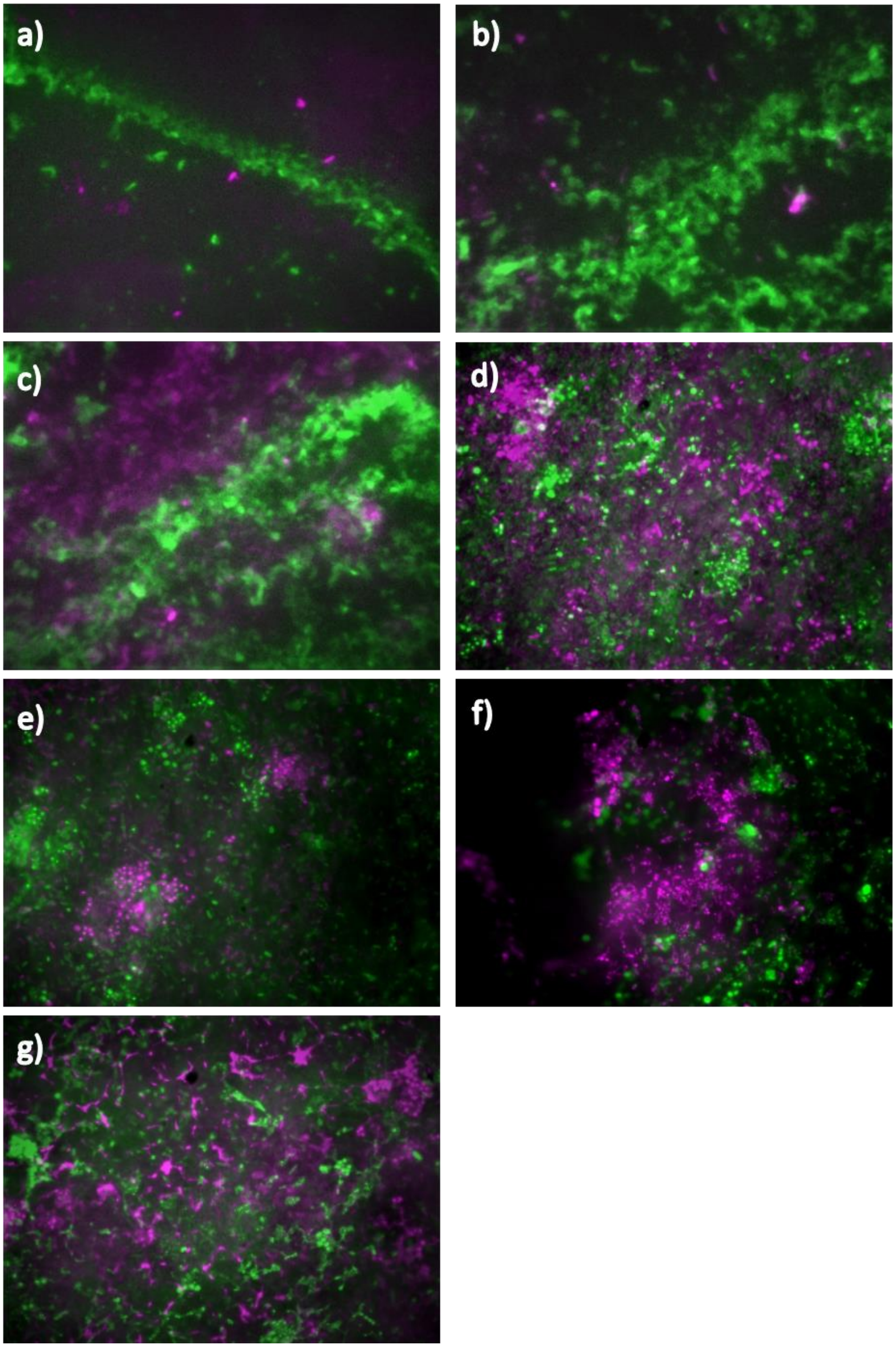

Supplementary Figure 4. Example images of the biofilm (EPS green and cells purple) from EVOS FL Auto 2 cell imaging system using a 100X oil immersion objective lens. They were selected at random from the images collected from slides on the middle section of the channel walls for a range of different flow regimes: **a)** Steady Flow (SF), channel 4, experiment 1; **b)** Small Vortex Flow (SVF), channel 9,

experiment 1; **c)** Large Vortex Flow (LVF), channel 1, experiment 1; **d)** LVF, channel 3 , experiment 1; **e)** High Shear Flow 1 (HSF1), channel 5 experiment 2; **f)** HSF, Channel 7, experiment 2; **g)** Oscillating Flow (OF), channel 11, experiment 2.

Temperature (°C)	week 1	week 2	week 3	week 4
**First set of experiments**				
tap water	18.3 ± 0.5	17.7 ± 0.2	17.2 ± 0.5	17.1 ± 0.3
air	21.4 ± 03	20.8 ± 0.2	20.7 ± 0.1	20.8 ± 0.3
**Second set of experiments**				
tap water	18.4 ± 0.3	18.3 ± 0.2	17.9 ± 0.2	17.1 ± 0.3
air	21.6 ± 0.4	21.2 ± 0.3	20.6 ± 0.3	19.8 ± 0.3

Supplementary Table 1. Temperature of tap water and of air (°C) at all duration of experiments.

	Wet mass of biofilm (mg)	Dry mass of biofilm (mg)
**First set of experiments**		
Steady flow (SF)	31.6	22.2
Small Vortex Flow (SVF)	62.1	36.8
Large Vortex Flow (LVF)	94.2	48.9
**Second set of experiments**		
Large Vortex Flow (LVF)	106.8	66.5
High Shear Flow 1 (HSF1)	64.5	52.4
High Shear Flow 2 (HSF2)	54.9	34.8
Oscillating Flow (OF)	86	49.8

Supplementary Table 2. The wet and dry mass of the biofilm (mg) in the first and second set of experiments.
